# Closing Clostridium botulinum Group III Genomes Using Long-Read Sequencing

**DOI:** 10.1128/MRA.01364-20

**Published:** 2021-06-03

**Authors:** Cedric Woudstra, Tommi Mäklin, Yagmur Derman, Luca Bano, Hanna Skarin, Christelle Mazuet, Antti Honkela, Miia Lindström

**Affiliations:** a Department of Food Hygiene and Environmental Health, University of Helsinki, Helsinki, Finland; b Department of Mathematics and Statistics, University of Helsinki, Helsinki, Finland; c Microbiology Laboratory, Istituto Zooprofiilattico Sperimentale delle Venezie, Villorba, Italy; d National Veterinary Institute, Uppsala, Sweden; e National Reference Center for Anaerobic Bacteria and Botulism, Institut Pasteur, Paris, France; f Department of Computer Science, University of Helsinki, Helsinki, Finland; Indiana University, Bloomington

## Abstract

Clostridium botulinum group III is the anaerobic Gram-positive bacterium producing the deadly neurotoxin responsible for animal botulism. Here, we used long-read sequencing to produce four complete genomes from Clostridium botulinum group III neurotoxin types C, D, C/D, and D/C. The protocol for obtaining high-molecular-weight DNA from C. botulinum group III is described.

## ANNOUNCEMENT

Clostridium botulinum group III strains producing botulinum neurotoxin (BoNT) types C and D and their mosaic forms, C/D and D/C, are responsible for animal botulism (1). With large outbreaks reported every year worldwide, this paralytic disease has a high economic impact on the farm industry (e.g., poultry, cattle). Losses in livestock production in Europe are roughly estimated at 90,000€ for a 60-head cattle herd or 20,000€ for a 30,000-bird poultry flock (2, 3). To date, only one closed genome is available as a reference on the NCBI public database (strain BKT015925 BoNT C/D, isolated in 2007 from a Swedish chicken farm; assembly accession number GCF_000204565.1). To understand the biology and epidemiology behind this pathogenic bacterium, more closed genome sequences are needed. In this report, a methodology for producing C. botulinum group III high-molecular-weight (HMW) DNA suitable for long-read sequencing is provided. This methodology was used to sequence the genome of four strains of C. botulinum group III, one of each toxin type, using PacBio single-molecule real-time (SMRT) sequencing. Detailed information related to the isolation of the strains is available in references 4 and 5.

Organisms were cultivated overnight from spore stock at 37°C under anaerobic conditions in 10 ml of tryptone-peptone-glucose-yeast (TPGY) medium (6). Fresh cultures were inoculated with 1/100 of the overnight cultures and incubated to reach exponential phase. Then, 1 ml of the cells was collected in Eppendorf tubes. Cells were centrifuged for 5 min at 6,000 rpm at 4°C, and the supernatant was discarded. The cell pellet was suspended in 80 μl of lysozyme (50 mg/ml), 80 μl of TE (10 mM Tris and 1 mM EDTA), and 2 μl of Triton 10% and incubated for 10 min at 37°C with shaking at 750 rpm in an Eppendorf thermomixer. Proteins were digested afterward by the addition of 1 μl of proteinase K (20 mg/ml), with 5 μl of EDTA (0.5 M), 35 μl of NaCl (3 M), and 25 μl of SDS (10%) added separately. After brief vortexing, the tube was incubated at 65°C for 5 min with shaking at 750 rpm and then cooled on ice for 5 min. Proteins were precipitated by the addition of 125 μl of ammonium acetate (7.5 M) and vortexed briefly before being placed on ice for 5 min. Cell debris and precipitated proteins were pelleted by centrifugation at maximum speed for 5 min at 4°C. Then, 300 μl of isopropanol was added to the supernatant, and DNA was precipitated by flipping the tube 40 times. This resulted in a characteristic white cloud precipitation containing HMW DNA. HMW DNA was pelleted by centrifugation at 3,500 rpm for 1 min at 4°C. The DNA pellet was then washed twice with 500 μl of ethanol 70% by inverting the tube 10 times, centrifuging it at 3,500 rpm for 1 min between each wash. The DNA pellet was then air-dried for 15 min and suspended in 100 μl of Tris 10 mM (pH 8) containing 1 μl of RNase A (10 mg/ml). The DNA was then stored at −20°C. Next, the DNA was sent to Macrogen, Inc., for sequencing. The libraries were prepared using the PacBio DNA template prep kit v2.0 according to the manufacturer’s protocol. Briefly, DNA was sheared using a Covaris platform and size selected for 10 kbp with AMPure PB beads (procedure details available from PacBio). Sequencing was performed using PacBio DNA/polymerase binding kit P5 on a PacBio RS II instrument. Illumina sequencing was performed at the Institute for Molecular Medicine Finland (FIMM). Libraries were prepared using a ThruPLEX DNA-seq kit (TaKaRa Bio, Inc., Japan), and sequencing was performed on a HiSeq 2500 platform (Illumina, CA, USA) using the paired-end kit v4 PE101 (Illumina). Raw reads were trimmed (minimum length, 35 bp; quality score, 0.03) using the CLC Genomics Workbench v10.1.1 with default parameters (Qiagen, Germany). Genomes were assembled using HGAP3 in SMRT Portal v2.2.0 and polished using Illumina reads with the CLC Genomics Workbench v10.1.1 with default parameters. The PacBio read *N*_50_ values and numbers of reads as well as the numbers of reads for the Illumina data are provided in Table 1. Chromosome sequences were set to start from replication initiator protein DnaA. Sequences of the bacteriophage p1 encoding BoNT locus were set to start and end at the terminal direct repeats (7). The sequences were automatically annotated with the National Center for Biotechnology Information (NCBI) Prokaryotic Genome Automatic Annotation Pipeline (PGAAP).

**TABLE 1:** NCBI accession numbers and assembly metrics of C. botulinum group III closed genomes

Strain	Provenance	Sample origin	Yr	BoNT type	No. of contigs	Chromosome size (Mbp)	G+C content (%)	No. of coding sequences (PGAAP)	NCBI accession no.	PacBio read *N*_50_ (bp)	No. of PacBio reads	PacBio sequencing depth (×)	PacBio SRA accession no.	Illumina sequencing depth (×)	Illumina SRA accession no.
Stockholm	Sweden	Mink	1946	C	6	2.30	27.98	2,558	CP063816.1–CP063821.1	19,590	108,543	400	SRX9781309	65	SRX10167271
1873	South Africa	Ham	1958	D	7	2.20	27.94	2,377	CP063822.1–CP063828.1	23,873	111,719	500	SRX9781308	600	SRX10168201
BKT2873	Sweden	Chicken	2007	C/D	4	2.84	28.57	2,867	CP063965.1–CP063968.1	17,137	82,860	250	SRX9781307	200	SRX487342
3859/11	Italy	Bovine	2011	D/C	6	2.88	28.52	3,094	CP063959.1–CP063964.1	25,670	103,674	300	SRX9781310	450	SRX10168202

**FIG 1:**
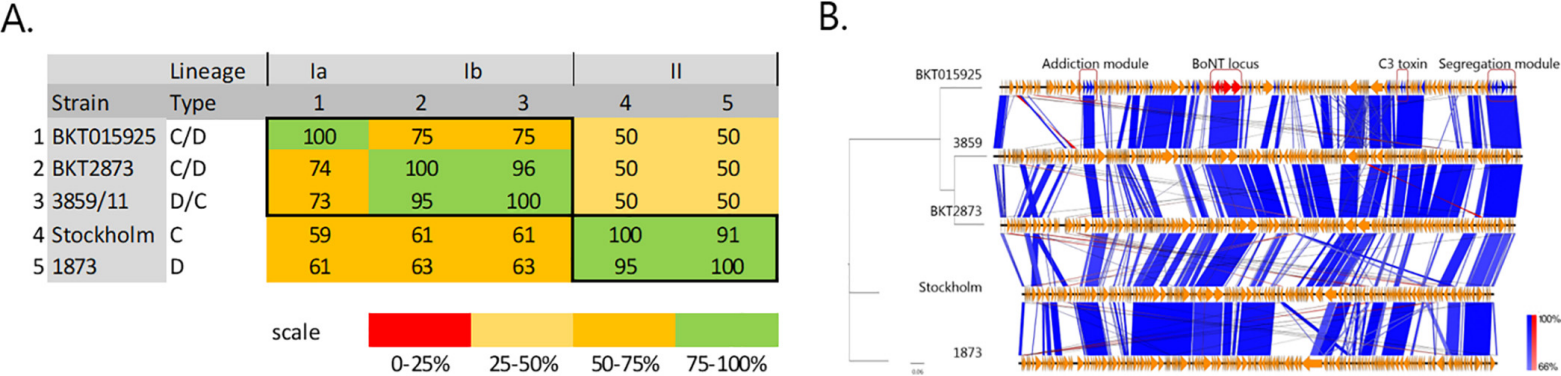
C. botulinum group III complete genome comparison. (A) Comparison of chromosome sequences from the four sequenced strains against the reference strain BKT015925. The comparison was done using Gegenees software v2.2.1 (8) in an all-against-all BLASTN whole-genome comparison with fragment length of 200 bp and step size of 100 bp. (B) Alignment of bacteriophage p1 containing the BoNT locus from the four sequenced strains against the reference strain BKT015925. The alignment was done using Easyfig v2.2.3 (11) with the BLAST option for the maximum E value at 0.001. Core genes in p1 from strain BKT05925 (obtained using the EDGAR platform [12]) are colored blue, and the BoNT locus is colored red.

Four closed genomes of C. botulinum group III were obtained (Table 1). While comparing the size and G+C content of each chromosome (Table 1), it clearly appeared that the strains clustered into two distinct clades. Strains Stockholm and 1873 grouped together but were distinct from strains BKT2873 and 3859/11. Comparison of the strain Stockholm, 1873, BKT2873, and 3959/11 chromosome sequences using Gegenees software v2.2.1 (8), together with chromosomes of reference strain BKT015925, confirmed the strain clustering, in accordance with previously published results (5) (Fig. 1A). Interestingly, when comparing the BoNT bacteriophage p1, alignment of the coding sequences (CDS) revealed a mosaic structure with only 38 genes as part of the core genome (Fig. 1B). In particular, core genes include an addiction module, the BoNT locus, the C3 toxin, phage proteins, and the TubZ plasmid segregation module (9, 10) (Fig. 1B). A more comprehensive analysis of C. botulinum group III BoNT bacteriophage p1 will be released in the near future.

### Data availability.

The annotated closed whole-genome sequences of the C. botulinum group III strains sequenced here were deposited in GenBank under the accession numbers reported in Table 1. The versions described in this paper are the first versions. Raw PacBio and Illumina data are available in the SRA (Table 1).
